# Reproducible Analysis of Post-Translational Modifications in Proteomes—Application to Human Mutations

**DOI:** 10.1371/journal.pone.0144692

**Published:** 2015-12-14

**Authors:** Alex S. Holehouse, Kristen M. Naegle

**Affiliations:** 1 Division of Biology and Biomedical Sciences, Washington University, St. Louis, MO, United States of America; 2 The Center for Biological Systems Engineering, Washington University, St. Louis, MO, United States of America; 3 Biomedical Engineering, Washington University, St. Louis, MO, United States of America; Swiss Institute of Bioinformatics, SWITZERLAND

## Abstract

**Background:**

Protein post-translational modifications (PTMs) are an important aspect of protein regulation. The number of PTMs discovered within the human proteome, and other proteomes, has been rapidly expanding in recent years. As a consequence of the rate in which new PTMs are identified, analysis done in one year may result in different conclusions when repeated in subsequent years. Among the various functional questions pertaining to PTMs, one important relationship to address is the interplay between modifications and mutations. Specifically, because the linear sequence surrounding a modification site often determines molecular recognition, it is hypothesized that mutations near sites of PTMs may be more likely to result in a detrimental effect on protein function, resulting in the development of disease.

**Methods and Results:**

We wrote an application programming interface (API) to make analysis of ProteomeScout, a comprehensive database of PTMs and protein information, easy and reproducible. We used this API to analyze the relationship between PTMs and human mutations associated with disease (based on the ‘Clinical Significance’ annotation from dbSNP). Proteins containing pathogenic mutations demonstrated a significant study bias which was controlled for by analyzing only well-studied proteins, based on their having at least one pathogenic mutation. We found that pathogenic mutations are significantly more likely to lie within eight amino acids of a phosphoserine, phosphotyrosine or ubiquitination site when compared to mutations in general, based on a Fisher’s Exact test. Despite the skew of pathogenic mutations occurring on positively charged arginines, we could not account for this relationship based only on residue type. Finally, we hypothesize a potential mechanism for a pathogenic mutation on RAF1, based on its proximity to a phosphorylation site, which represents a subtle regulation difference that may explain why its biochemical effect has failed to be uncovered previously. The combination of the API and a dynamically expanding PTM database will make the reanalysis of this question and other systems-level questions easier in the future.

## Introduction

The development of high-throughput measurement technologies, such as mass spectrometry based proteomics, has led to a rapid expansion in the discovery of post-translational modifications (PTMs) [1]. In order to understand how these PTMs regulate protein turnover, activity, interactions, localization, and other aspects of protein function, a large body of research has developed based on the analysis of PTMs relative to other protein features [2–6] and the relationship between PTMs and protein evolution [7–12]. Several similar systems-level studies have also been focused on how the gain, loss, or dysregulation of PTMs may be involved in human disease [13–15]. To perform these studies, researchers have manually combined a variety of PTM resources—a process which poses a significant challenge from a data acquisition, scrubbing, and warehousing perspective. Beyond this, such an analysis is based on a single snapshot of a collection of resources, a perspective which does not easily allow a longitudinal analysis to explore how a relationships of interest may change as new information becomes available and the PTM datasets evolve. In this study we examine how the number of identified PTMs has changed over time, a result which suggests there could be serious limitations in the conclusions drawn from systems-level analysis of PTMs in previous years, when compared to analysis done using data available today.

**Fig 1:**
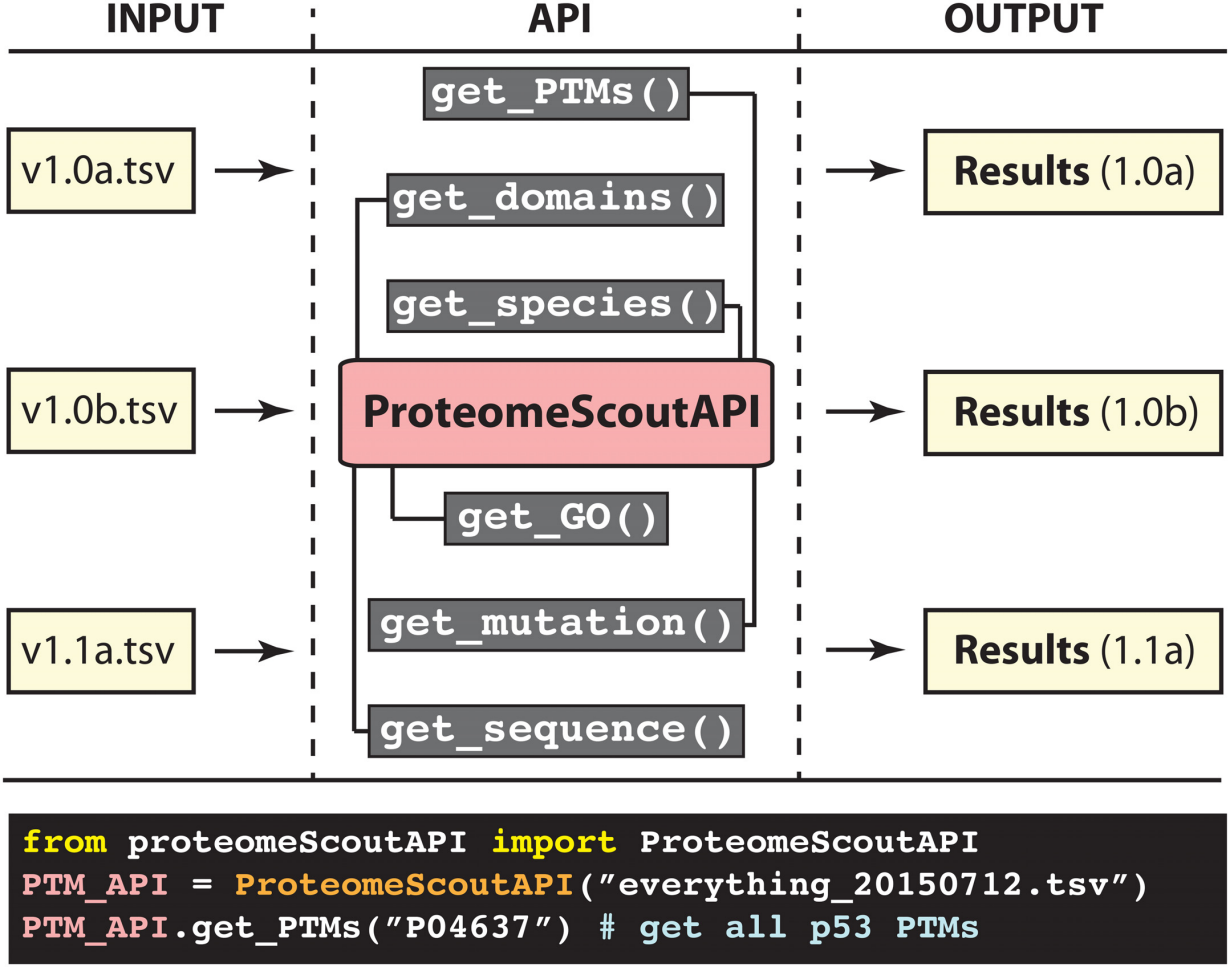
ProteomeScout API and its application to reproducible analyses. The API block gives examples of functions that operate on a tab-separated file, which can be downloaded from ProteomeScout [16]. The analysis that can be done on the PTM-centric information in ProteomeScout can take on many forms given the flexibility of the API. Example code is given for retrieving all PTMs associated with the protein p53 (UniProtKB accession P04637). As the ProteomeScout dataset evolves and grows through new external data the same analyses can be re-run in the future.

To overcome the challenge of data curation and enable easy updates and future re-analysis, we wrote an application program interface (API) for the expansive and dynamically growing database of PTMs, ProteomeScout [16] (Fig 1). The Python-based API makes it easy to interact with a ProteomeScout database download. The ProteomeScout database has a stable release every six months, as well as weekly updates to reflect dynamics snapshots of the current state of knowledge of PTMs across organisms. We have used this API, in conjunction with the current stable release of the ProteomeScout database, to analyze the relationship between PTMs and mutations. Specifically, we, and others [14], hypothesize that mutations within the recognition sequence of PTMs used by enzymes and binding partners would be likely to cause protein dysregulation and manifest as human-disease related mutations.

To test the hypothesis of whether disease-causing mutations, based on the ‘Clinical Significance’ category within dbSNP [17], are more likely to be near sites of PTMs, we found that we first had to correct for study bias. Proteins with at least one pathogenic mutation were consistently more likely to have other annotations, including PTMs and Gene Ontology terms [18]. Therefore, we first controlled for study bias by only analyzing the set of proteins with at least one pathogenic mutation. We found that pathogenic mutations were more likely to be within eight amino acids of a PTM of several types, including ubiquitination and phosphorylation of serine and tyrosine residues. The limited amount of data currently available on other modifications limits the analysis of these PTMs, although this may change in the future as the associated datasets grow as a result of community-based deposition of PTMs in ProteomeScout or the major compendia that ProteomeScout incorporates. Since pathogenic mutations were much more likely to occur on arginine residues, we tested for the possibility that PTMs and pathogenic mutations were coincidentally together based on surface accessibility. However, in control tests we could not explain the enrichment of PTMs near pathogenic mutations based on charge alone. Importantly, the API, the specific database snapshot that was used in this analysis, and the open-source analysis scripts, are available as supplementary information on our website, making the reproducibility of this analysis easy and certain. Additionally, this means that we and others can continuously update these proteome-wide statistical relationships between mutations and PTMs as the database expands and a new database file is used in the analysis pipeline.

After discovering that globally, the nearness of mutations to a PTM may be indicative of a likelihood of being related to disease, we asked whether we could use this to develop specific hypotheses of how pathogenic mutations may alter protein function. The serine/threonine kinase RAF1 is heavily mutated, and several of these mutations are linked to dysregulation of MAPK activity [19, 20]. Despite a link to disease, the RAF1 V263A mutation has failed to demonstrate disruption of 14-3-3 protein binding, which leads to increased RAF1 activity [21, 22], unlike other nearby mutations [23]. By understanding the PTMs within the region and using the data available on ProteomeScout from a quantitative phosphoproteomics study of regulation of this region during stem cell differentiation [24], we hypothesize that the V263A mutation may be affecting regulation important to differentiation, which results in the development of MAPK-related developmental disorders where V263A has been observed [19, 20]. These explorations demonstrate that the under-appreciation of nearby PTMs may be playing a role in RAF1 regulation, particularly during development, and explain how biochemical assays may have failed to uncover the effect of the V263A mutation via 14-3-3 misrecognition alone.

## Materials and Methods

### PTM growth

Numbers of PTMs for 1999 and 2004 were taken from early PhosphoBase and PhosphoSite papers, [25] and [26], respectively. For information from latter years, we parsed database downloads of files from PhosphoSite [27]. Database downloads were performed by the authors on the following dates: May 2007, September 2009, July 2013, January 2014, and July 2015. The data file and the iPythonNotebook that analyzed these data are available on ProteomeScout’s documentation page (https://www.assembla.com/spaces/proteomescout/wiki). The file of all PTMs and numbers is also available as S1 Table.

### Implementation of the ProteomeScout API and mutations analysis

The ProteomeScoutAPI was written in Python and is available in a Mercurial repository on the ProteomeScout Assembla project page. The current stable release (v1.0b, November, 2015) of ProteomeScout mammalian PTM file was downloaded from the ProteomeScout stable release FTP site ftp://ftp.seas.wustl.edu/pub/ProteomeScout_DbF/current_stable_release/. All calculations and analyses were performed in Python, specifically using iPython [28] notebooks and the following open-source projects: Pandas [29], NumPy [30], and Matplotlib [31]. Testing for enrichment was done using a one-sided Fisher’s Exact test. We counted amino acids uniquely for having either a mutation or a modification within the specified window of 0 (on the amino acid) or 8 (within +/- 8 amino acids of the residue). If more than one mutation exists on the same amino acid and at least one of the mutations was known to be pathogenic or disease-related, then we assigned that residue a pathogenic/disease phenotype during all analyses. False discovery rate [32] was used as a multiple hypothesis correction technique, where denoted.

In accordance with recommendations on best practices [33] for developing bioinformatics software, the ProteomeScoutAPI has a full software testing suite, built using the Python unittest framework. This series of tests ensures that updates and changes to the code do not inadvertently lead to the introduction of software bugs elsewhere. Importantly, extending and growing this suite to accommodate new features is simply a few lines of additional code, ensuring that as the ProteomeScoutAPI grows and new functionality is added, a formal testing framework can be built in parallel.

The analysis code is available on GitHub and can be visualized on nbviewer at: http://nbviewer.ipython.org/github/knaegle/MutationsNotebooks/tree/master/. All calculations and graphs for study bias, PTM enrichment, and resampling for charge distributions are available in the iPython notebooks. SVG exports for RAF1 studies and data from the study by Rigbolt et al. [24] were taken from ProteomeScout [16].

### Definition of disease mutations

This study focuses on mutations which were identified as being disease-related based on having a dbSNP Clinical Significance annotation of ‘pathogenic’. However, we also performed an identical analysis on mutations identified as being disease-related based on their UniProKB annotation, as defined by the Human polymorphisms and disease mutations index file (http://www.uniprot.org/docs/humsavar, S3 Table). The dbSNP annotations yield 784 proteins with at least one pathogenic mutation, while the UniProtKB annotations yield 2273 such proteins. The dbSNP annotations are taken directly from the ProteomeScout database annotations using the ProteomeScoutAPI. The UniProtKB annotations were pulled from the humsavar file and then mapped to ProteomeScout database records. All the code for performing this analysis is provided.

### Controlling for amino acid content of the pathogenic set

We created random foregrounds the same size and with the same distribution of amino acid types as the real foreground, the pathogenic set, which is enriched for arginines in particular. We then tested these random foregrounds for enrichment of nearby PTMs using the same analysis as outlined above. We ran many sets of 10 random foregrounds and never observed enrichment between mutations in random foregrounds and PTMs above what was expected by random chance alone.

## Results and Discussion

### Growth of the PTM proteome (PTMome)

**Fig 2:**
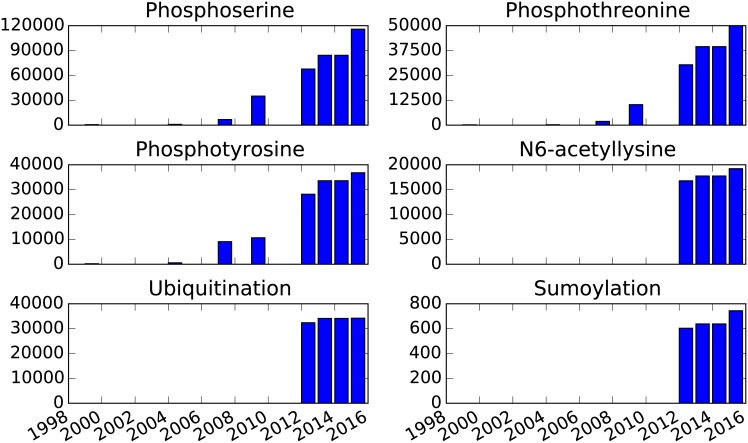
Growth of identified human PTMs across time. Data is based on PhosphoBase publications in 1999 [25] and 2004 [26] and PhosphoSite [27] downloads from 2007, 2009, 2012, 2013, 2014, and 2015.

Our understanding of post-translational modifications in a wide range of organisms is rapidly expanding. We explored the growth of the most well-studied PTMs in the human proteome by looking at a single resource of PTMs across time, shown in Fig 2. Specifically, we examined the PhosphoSite database [27], obtaining the number of phosphosites from the PhosphoSite papers in 1999 [25] and 2004 [26] as well as the datasets downloaded by the PTMScout and ProteomeScout authors from PhosphoSite between 2007 and 2015. The number of identified phosphosites grows exponentially during this decade and a half, with just tens of sites in 1999 growing to thousands of known sites by 2015. The first acetylation, sumoylation, and ubiquitination database entries appear in 2010, when high-throughput purification methods were developed and tied to mass spectrometry [1]. The slowing of growth in the number of newly identified phosphorylation sites in the latter years may indicate that the number of novel phosphorylation sites discovered in human proteins from standard cell lines is approaching saturation. However, it is expected that the identification of phosphosites in new organisms, as well as tissue- and disease-specific sites, will take over as the primary contributors to novel measurements in the coming years. While phosphorylation has, historically, been the most well understood class of PTMs, coverage of other modification types may be expected to grow robustly based on these trends.

The growth of information associated with PTMs—as well as the surrounding information regarding protein sequence annotations—motivates the necessity for a sustainable database of post-translational modifications and protein annotations such that analysis and research can be updated easily. Certainly, these results indicate that conclusions drawn as recently as 2010 would no longer reflects our understanding in 2015, and that the shift in our understanding based on newly available data alters the set of relevant questions surrounding PTMs. In the remainder of this work we will introduce our solution to performing reproducible analyses of the PTMome and use this tool to explore the relationship between PTMs and human mutations.

### Reproducible analysis of PTM-centric studies

To address the challenge of a rapidly changing PTMome, we built an application programming interface (API) to interact with ProteomeScout database files. ProteomeScout’s database files are updated weekly and every six months a stable release is created. The weekly updates may include changes since the last week as a result of users uploading data. Uploading data triggers an update of annotations associated with those specific protein records in the database. In the stable release, all protein annotations, such as Gene Ontology terms [18] and mutations [17, 34] are updated. Additionally, the major compendia, such as UniProtKB and Phosphosite PTM datasets are updated. The weekly releases are available directly on the ProteomeScout website and the stable releases are available via FTP hosting, as described in the methods section.

In flat text files generated by ProteomeScout, all of the annotations for proteins are written in column-wise fashion with multiple annotations of a certain type being separated by semicolons. The database files are described in detail on ProteomeScout’s wiki (available at https://www.assembla.com/spaces/proteomescout/wiki). However, the ProteomeScoutAPI removes the necessity of understanding the exact formatting of the database file by automatically parsing the file into Python objects that can be interacted with in a straightforward manner. For example, using the get_mutations(<ACC>) command, one can retrieve all mutations for a protein record, based on a particular protein accession. The ProteomeScoutAPI code and help documentation are available on the freely-hosted ProteomeScout project page (https://www.assembla.com/spaces/proteomescout). Fig 1 demonstrates the usage of the API and how, when analyses are based on a ProteomeScout database download, the analysis written in Python with the API can be used for the re-analysis of the data with the same database download or updated with future database downloads to reflect the growing knowledge of PTM and other protein information.

### Study bias confounds proteome analysis

In this study, we wish to test the hypothesis that disease-related point mutations are more likely to be near or at sites of post-translational modifications. To do this we used the non-synonymous mutation annotations within ProteomeScout, which are harvested from NCBI’s dbSNP [17]. A subset of these mutations are annotated with ‘Clinical Significance’, which includes the categories ‘Pathogenic’ and ‘Non-pathogenic’. Prior to testing mutations from all protein records for their proximity to known PTMs, we first explored for the possibility of study bias, i.e. the possibility that proteins with pathogenic mutations are more likely to have annotations of other types simply because they are well studied.

**Fig 3:**
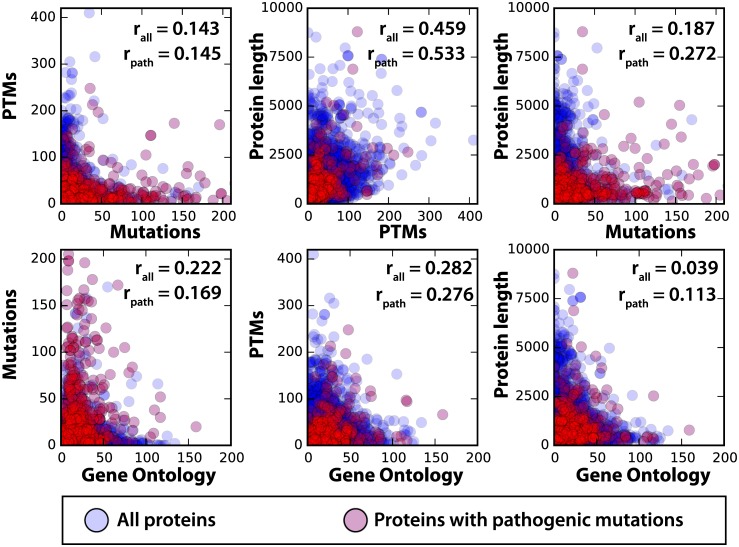
Correlation between protein annotations. Scatter plots for all comparisons of the number of annotations per protein or the length of the protein. Points in blue represent the number of annotations on a protein that does not contain a pathogenic mutation. Red represents a protein with at least one pathogenic mutation, which becomes the set of proteins studied in subsequent analyses. Correlations between numbers of labels on a per protein basis are given as well as the correlation between annotations on the pathogenic set. All correlations were significant with a p-value less than 1E-08. These plots and correlation values, broken down by PTM type, are available in S1 Fig.

To test for study bias, we first looked at the correlation between the number (per protein) of two different protein annotations, or the number of protein annotations versus the total length of the protein (Fig 3). Specifically, the annotations we considered were GO terms, mutation records, and PTM records. In total, 21,910 human protein records existed in the stable release considered for this study, 15,480 of which have at least one known mutation. The p-values of all correlation values are significant, and there is some degree of positive correlation between every pairing. The highest correlation exists between PTMs and sequence length, indicating that the longer a sequence is, the more likely it is to have a larger number of PTMs. To a lesser extent this also holds true for mutations.

The positive correlation of all mutations with PTMs and with pathogenic mutations and PTMs (Fig 3) led us to test for specific study bias of proteins containing records of pathogenic mutations. To test for this, we performed a Fisher’s Exact Test to compare the significance of label distributions based on proteins having at least one pathogenic mutation. There were 784 proteins with a pathogenic mutation and these proteins are significantly more likely to have a large number of GO terms and PTMs in addition to having many annotated mutations in general. For example, when we tested for enrichment of proteins having at least ten GO annotations or PTMs we find pathogenic mutation containing proteins to be significantly enriched (p-values of 2e-101 and 2e-19, respectively). Study bias appears to occur on the level of the full protein, i.e. a protein with a known pathogenic mutation is much more likely to have more GO annotations, PTM annotations, and mutations in general. Therefore, in the following work we control for study bias by only considering those proteins that have at least one known pathogenic mutation, and are therefore more likely to have more annotations and mutations overall.

### Pathogenic mutations are enriched for nearby PTMs

To test for the relationship between mutations and PTMs, controlling for study bias, we used the set of human proteins that have at least one pathogenic mutation. This included 795 human proteins, which all together contain 21,085 mutations (1,896 are pathogenic) and 16,701 total PTMs. For each of the human PTMs with a sufficiently large number of annotations (phosphoserine, phosphothreonine, phosphotyrosine, acetylation, ubiquitination, and N-Glycosylation), we tested for the significance of having a pathogenic mutation on the site of modification or within a possible recognition area of the site of modification using a one-sided Fisher’s Exact test. We found that pathogenic mutations were not significantly more likely occur on sites of modification than other sites in the proteome. Only about 10% of all mutations (1,896) occur on a specific site of modification with relatively small numbers for the individual modification types. Therefore, we cannot rule out that lack of significance is not meaningful based on the sample sizes. However, most modifications demonstrated a significant relationship with pathogenic mutations when a regulatory window was considered (Table 1). In particular, ubiquitination occurring near a site of mutation means the mutation is much more likely to have a known relationship with human disease, whereas N6-acetlylysine and N-Glycosylation were not significantly related to pathogenic mutations (possibly also due to limited set size). To a lesser extent than ubiquitination, phosphorylation of serines, threonines and tyrosines were significantly related to pathogenic mutations. These results indicate there is a strong relationship between the likelihood that mutations will be associated with pathogenicity and their proximity to known protein modifications, and that this relationship is dependent on the type of modification.

**Table 1:** Significance between dbSNP pathogenic or disease mutations and PTMs.

**Modification**	**dbSNP ‘pathogenic’ p-value**	**UniProtKB ‘Disease’ p-value**
Ubiquitination	2E-07	4E-03
Phosphotyrosine	3E-02	1E-05
Phosphoserine	2E-02	1E+00
Phosphothreonine	2E-02	1E+00
N6-acetyllysine	3E-01	1E-01
N-Glycosylation	1E+00	7E-01

Significance calculated on one-sided Fisher’s exact test for ‘pathogenic’ or ‘disease’ mutations having the designated PTM within eight amino acids, compared to all mutations in the background set (corrected for study bias).

The relationship between modifications and pathogenic mutations indicates that misrecognition of PTM sites by enzymes or binding domains could lead to functional disruption which is more likely to cause disease than other mutations in general. However, an alternate hypothesis is that pathogenic mutations and sites of modification are coincident based on some other property they have in common. For example, if mutations on the protein surface are more likely to be detrimental and modifications are more likely to occur on the surface of the protein, then their coincidental location on the protein alone might explain the significant relationship we observed. Indeed, we found a skew in the types of amino acids that have known pathogenic mutations. Specifically, there is a significant bias towards arginines in the pathogenic set of mutations (p-value 2E-15), which might indicate a higher likelihood of surface accessibility. To rule out the effect of the distribution of amino acid types, and possible location of amino acids within a protein structure, we created random foregrounds that contained the same number of mutations as the pathogenic set and the same distribution of amino acids. In ten randomized trials, we never observed a significant enrichment of these foregrounds having nearby modifications. These results indicate that globally there is a significant relationship between pathogenic human mutations and modifications that cannot be accounted for by co-incidental charge of the mutations alone.

### Extending to a larger set of disease-related mutations

We used dbSNP’s ‘pathogenic’ mutations for our initial tests since these labels are available in the ProteomeScout database. However, there is a significantly larger number of ‘Disease’-related variants available in the UniProtKB database. Therefore, we also tested the relationship between the likelihood of being labeled as disease-related in UniProtKB [35] with PTMs using the same analysis by cross-referencing from this set to the ProteomeScout database file using the API. We observed similar trends with regards to study bias—disease-mutation containing proteins were much more likely to have more Gene Ontology terms and PTMs. Therefore, we controlled for study bias in this set in the same manner, by creating a background consisting of only the mutations from proteins that contain at least one disease-annotated mutation. Of the 68,819 mutations in UniProtKB, 21,999 are labeled with the annotation ‘Disease’ (32%). However, when we identified the subset of proteins with at least one disease-labeled mutation and re-evaluate the annotation associated with mutations in that subset of proteins the proportion of disease mutations goes from 32% to 68%. This suggests two possibilities: 1) certain proteins are hubs for pathogenicity or 2) there is bias in the annotation of disease mutations. In this second “rich-get-richer” scenario, the identification of a disease-related mutation on a protein may lead to focused efforts on identification of other mutations on that protein that also result in a disease annotation. There tends to be clustering of disease annotated mutations on proteins (S3 Table), which supports both hypotheses.

Despite the inherent differences of the two mutation datasets, with regard to their size and distribution of pathogenic/disease to total mutation ratio (68% vs. 10%), we performed the same statistical analysis of testing for enrichment of disease mutations set near known PTMs (Table 1). Ubiquitination and tyrosine phosphorylation continue to be enriched—they are much more likely to be near a disease-related mutation than would be expected by random chance. However, there is a change in their significance, relative to the findings in the dbSNP pathogenic set and no other modifications are enriched. Considering this, the exact conclusions are highly dependent on the set of mutations and their disease annotations. As a corollary, these results are also dependent on the current state of PTM knowledge. In particular, most PTMs such as sumoylation, myristoylation, and methylation, have too few annotations for sufficient statistical testing. As PTM measurement and discovery expands, particularly for those PTMs with few ProteomeScout annotations, we (and others) can test for a relationship with disease-related mutations in the future. The dependence on the data of mutations used and state of PTM knowledge on the findings highlights the importance for a framework that enables the easy reproducibility of current results, extensibility to different analysis types, and the re-analysis at a future date when knowledge has changed.

### Using PTMs to predict the effect of mutations

**Fig 4:**
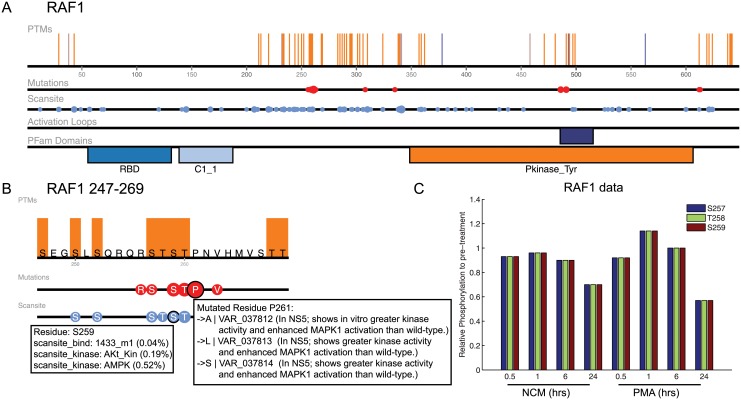
Predicting effect of RAF1 mutations. (A) Full length RAF1 from ProteomeScout [16] with PTM annotations, domains, mutations from dbSNP, and Scansite predictions. (B) RAF1 in the area of interest near S259, which is involved in 14-3-3 recognition. (C) Quantitative measurements of phosphorylation on S257, T258 and S259 from a study of human embryonic stem cells without conditioning and with conditioning for stem cell differentiation (PMA treatment) [24]. Data from study downloaded from ProteomeScout [16].

Given that there is consistent insight to pathogenicity based on proximity to PTMs, we sought to identify whether knowing the proximity or number of modifications near a mutation could be helpful in determining the impact of a mutation. S2 Table lists the sites of human dbSNP mutations. Not surprisingly, 21 of the top 28 mutations with the largest number of nearby modifications are on the tumor suppressor protein TP53. Also, in the top 100 of mutations, ranked by number of nearby modifications, are a series of RAF1 mutations occurring between positions R256 and V263, Fig 4A and 4B. When phosphorylated, S259 is recognized by 14-3-3, whose binding negatively regulates RAF1 activity and subsequently decreases MAPK activation [21, 22]. Multiple genetic studies have identified RAF1 mutations near S259 in patients with Noonan and LEOPARD Syndrome, both syndromes which show a characteristic increase in MAPK activation [19, 20]. Multiple independent studies have verified that a number of these mutations reduce binding to 14-3-3 and increase MAPK activation, including R256S, S257L, S259F, T260I/R, and P261A/S/L [19, 20, 23]. Based on these results, it is clear that these disease-related mutations act through the disruption of the recognition and regulation of PTMs and highlights the utility of understanding the relationship between mutations and modifications.

The ProteomeScout protein view [16] of this region of RAF1 highlights the density of phosphorylation sites and known mutations. In addition to S259 phosphorylation, which has a well-understood role in regulating RAF1 activity, phosphorylation has been observed on other nearby phosphorylation sites including S257, T258, and T260, Fig 4A. ProteomeScout currently includes one experiment with quantitative measurements of phosphorylation in this region. Rigbolt et al. identified phosphorylation of S257, T258, and S259 in human embryonic stem cells (hESCs) and quantitatively measured their relative phosphorylation levels in response to differentiation. Fig 4C contains the plot of ProteomeScout data from [24] for this region of phosphorylation sites in changing to non-conditioned media (NCM) or in response to phorbol 12-myristate 13-acetate (PMA), treatments that initiate stem cell differentiation, compared to the pre-treated samples. The three sites in the region of interest, which are in the concentrated region of mutations found in Noonan and LEOPARD syndrome samples, follow the same pattern and exhibit no relative change until after 24 hours of treatment, where they decrease in phosphorylation. We were surprised the quantitative data amongst the three sites were identical. Upon revisiting the original supplementary information on this dataset in Rigbolt et al., the data is faithfully represented in ProteomeScout, but the assignment score suggests the specific site of modification was not accurately identified. However, given multiple sources of identification for these phosphorylation sites [23, 27, 35–43] it is likely that this experiment indicates that at least in hESCs, phosphorylation occurs on some subset of these sites and that they demonstrate a dynamic response to initiation of stem cell differentiation. Phosphorylation on these alternate sites is not currently appreciated as playing a role in regulating 14-3-3 activity, yet this may represent a process by which traditional S259/14-3-3 recognition is altered. It also expands the possibility of mechanisms by which these mutations affect protein function and regulation, and may help lead to hypotheses of how V263A plays a role in development of Noonan Syndrome, despite having no measurable effect on 14-3-3 binding [23].

## Supporting Information

S1 Table The number of all modifications collected from PhosphoSite from 1998 to 2015. (CSV)

S2 Table List of human mutations and PTMs from dbSNP. (XLSX)

S3 Table List of human mutations and PTMs from UniProtKB. (TXT)

S1 Fig Correlation plots and values for all metrics broken down by PTM type. (PDF)
