# Supplementary material for: Reproducible Analysis of Post-Translational Modifications in Proteomes—Application to Human Mutations
Source: PLoS One. 2015 Dec 14;10(12):e0144692. doi: 10.1371/journal.pone.0144692 (PMC4685989; doi:10.1371/journal.pone.0144692)
Supplement: S1 Fig — (PDF) [file pone.0144692.s004.pdf]

dbSNP:Ubiquitination

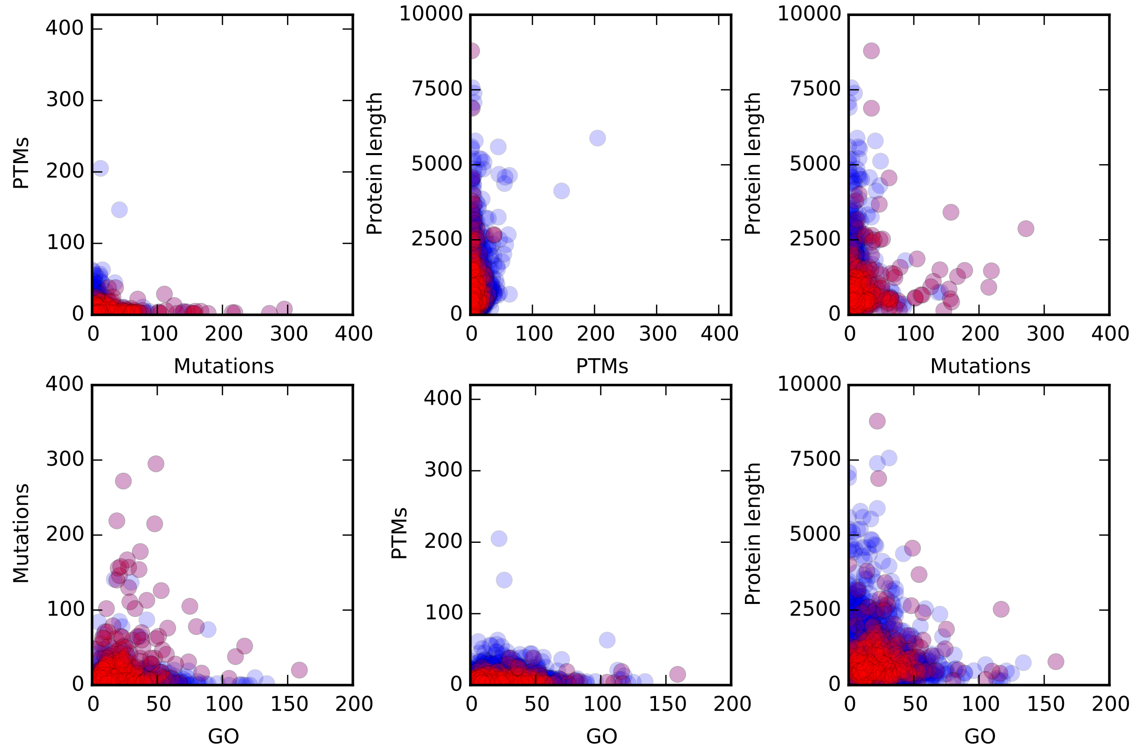

All

GO and Mutations have correlation	0.19	with pvalue of	5E-45
GO and Sequence have correlation	0.01	with pvalue of	5E-01
GO and Ubiq have correlation	0.23	with pvalue of	5E-67
Mutations and Sequence have correlation	0.19	with pvalue of	4E-49
Mutations and Ubiq have correlation	0.01	with pvalue of	5E-01
Sequence and Ubiq have correlation	0.17	with pvalue of	1E-39

Pathogenic

GO and Mutations have correlation	0.19	with pvalue of	2E-03
GO and Sequence have correlation	0.08	with pvalue of	2E-01
GO and Ubiq have correlation	0.11	with pvalue of	8E-02
Mutations and Sequence have correlation	0.18	with pvalue of	3E-03
Mutations and Ubiq have correlation	-0.06	with pvalue of	4E-01
Sequence and Ubiq have correlation	0.07	with pvalue of	2E-01

dbSNP: Phosphotyrosine

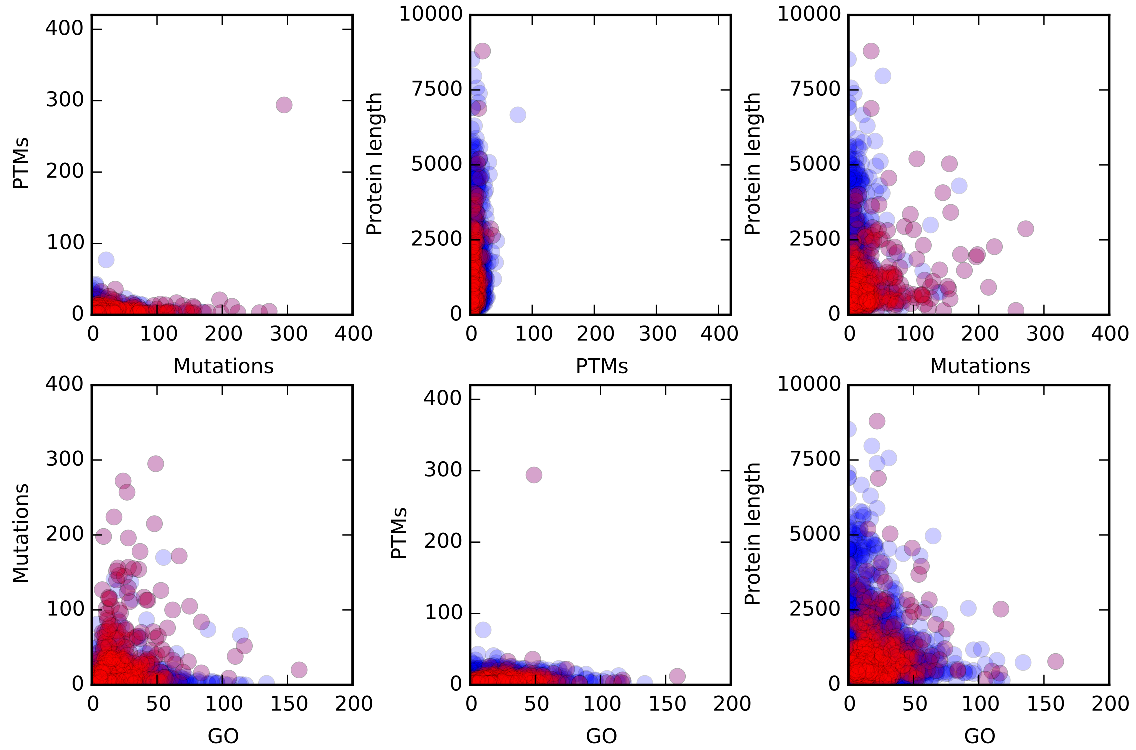

All

GO and Mutations have correlation	0.21 with pvalue of 3E-75
GO and Sequence have correlation	0.01 with pvalue of 6E-01
GO and pTyr have correlation	0.20 with pvalue of 6E-73
Mutations and Sequence have correlation	0.21 with pvalue of 1E-78
Mutations and pTyr have correlation	0.10 with pvalue of 1E-18
Sequence and pTyr have correlation	0.33 with pvalue of 2E-191

Pathogenic

GO and Mutations have correlation	0.19 with pvalue of 9E-05
GO and Sequence have correlation	0.11 with pvalue of 2E-02
GO and pTyr have correlation	0.23 with pvalue of 4E-06
Mutations and Sequence have correlation	0.27 with pvalue of 4E-08
Mutations and pTyr have correlation	0.09 with pvalue of 8E-02
Sequence and pTyr have correlation	0.44 with pvalue of 2E-20

dbSNP:Phosphoserine

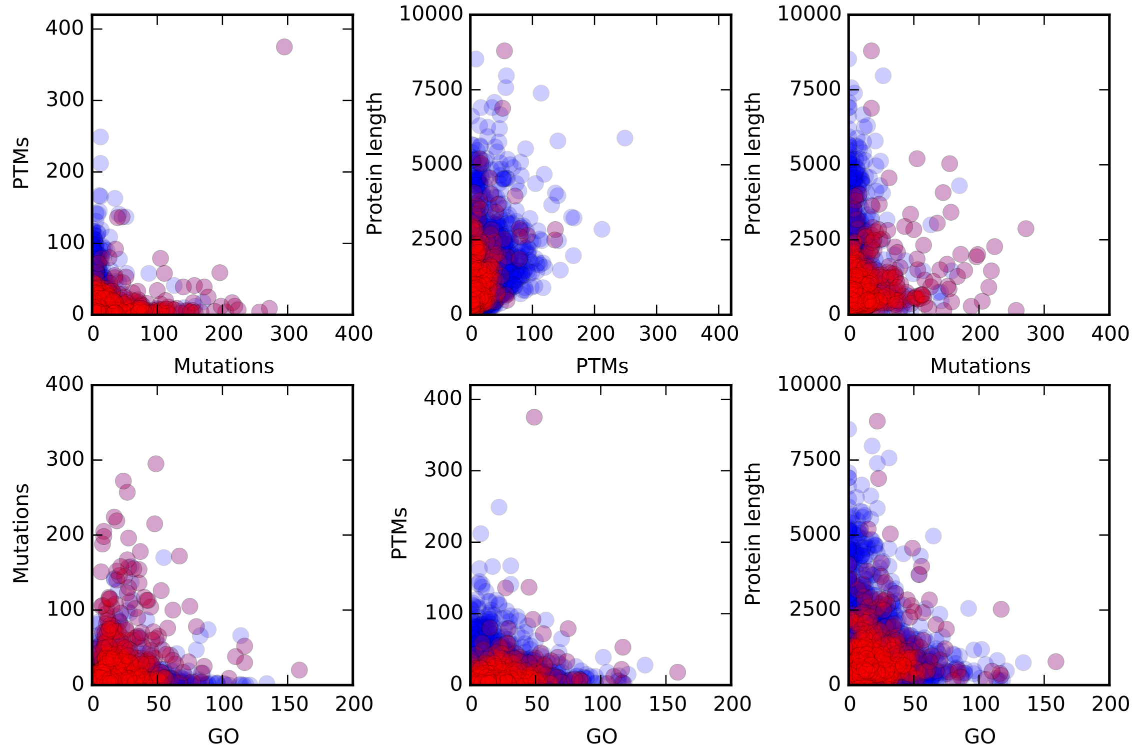

All

GO and Mutations have correlation 0.21 with pvalue of 7E-144
GO and Sequence have correlation 0.01 with pvalue of 2E-01
GO and pSer have correlation 0.12 with pvalue of 5E-49
Mutations and Sequence have correlation 0.19 with pvalue of 5E-11
Mutations and pSer have correlation 0.08 with pvalue of 1E-20
Sequence and pSer have correlation 0.46 with pvalue of 0E+00

Pathogenic

GO and Mutations have correlation 0.17 with pvalue of 7E-05
GO and Sequence have correlation 0.09 with pvalue of 4E-02
GO and pSer have correlation 0.23 with pvalue of 2E-08
Mutations and Sequence have correlation 0.26 with pvalue of 3E-10
Mutations and pSer have correlation 0.09 with pvalue of 3E-02
Sequence and pSer have correlation 0.48 with pvalue of 6E-34

dbSNP:Phosphothreonine

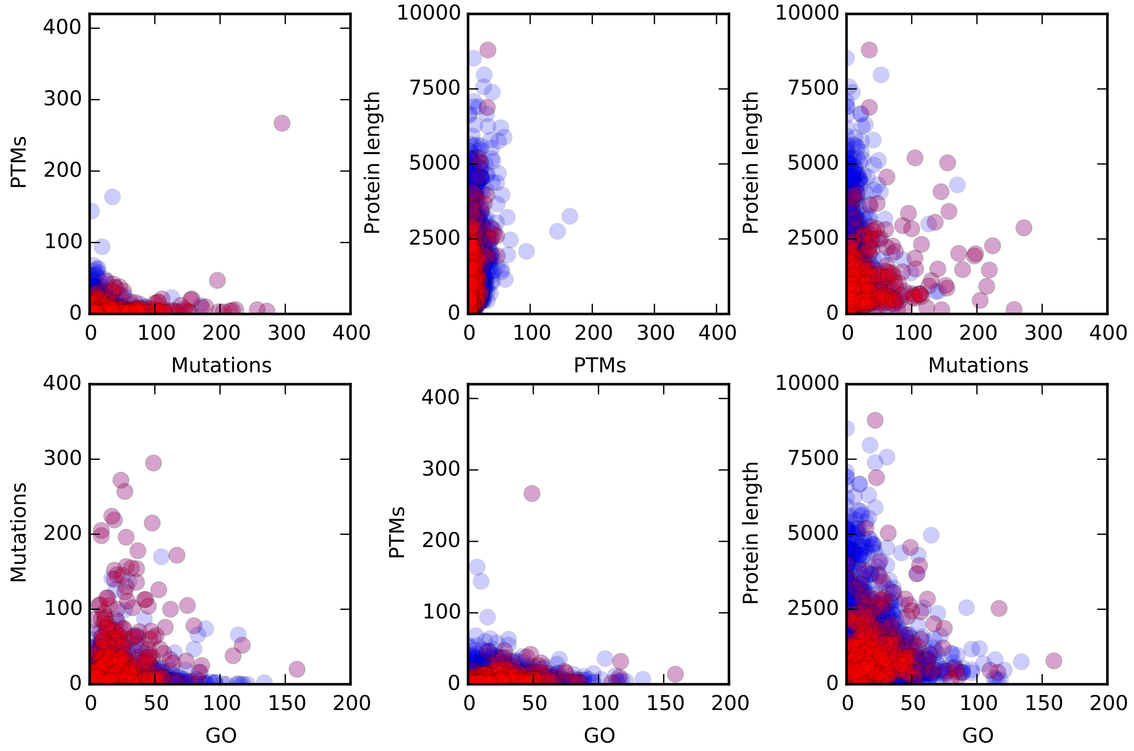

All

```
GO and Mutations have correlation         0.21 with pvalue of 1E-98
GO and Sequence have correlation         -0.00 with pvalue of 9E-01
GO and pThr have correlation              0.14 with pvalue of 2E-42
Mutations and Sequence have correlation  0.20 with pvalue of 2E-92
Mutations and pThr have correlation       0.10 with pvalue of 1E-22
Sequence and pThr have correlation        0.44 with pvalue of 0E+00
```

Pathogenic

```
GO and Mutations have correlation         0.17 with pvalue of 3E-04
GO and Sequence have correlation          0.09 with pvalue of 8E-02
GO and pThr have correlation              0.26 with pvalue of 3E-08
Mutations and Sequence have correlation   0.26 with pvalue of 5E-08
Mutations and pThr have correlation       0.11 with pvalue of 2E-02
Sequence and pThr have correlation        0.54 with pvalue of 8E-34
```

dbSNP:N6-acetyllysine

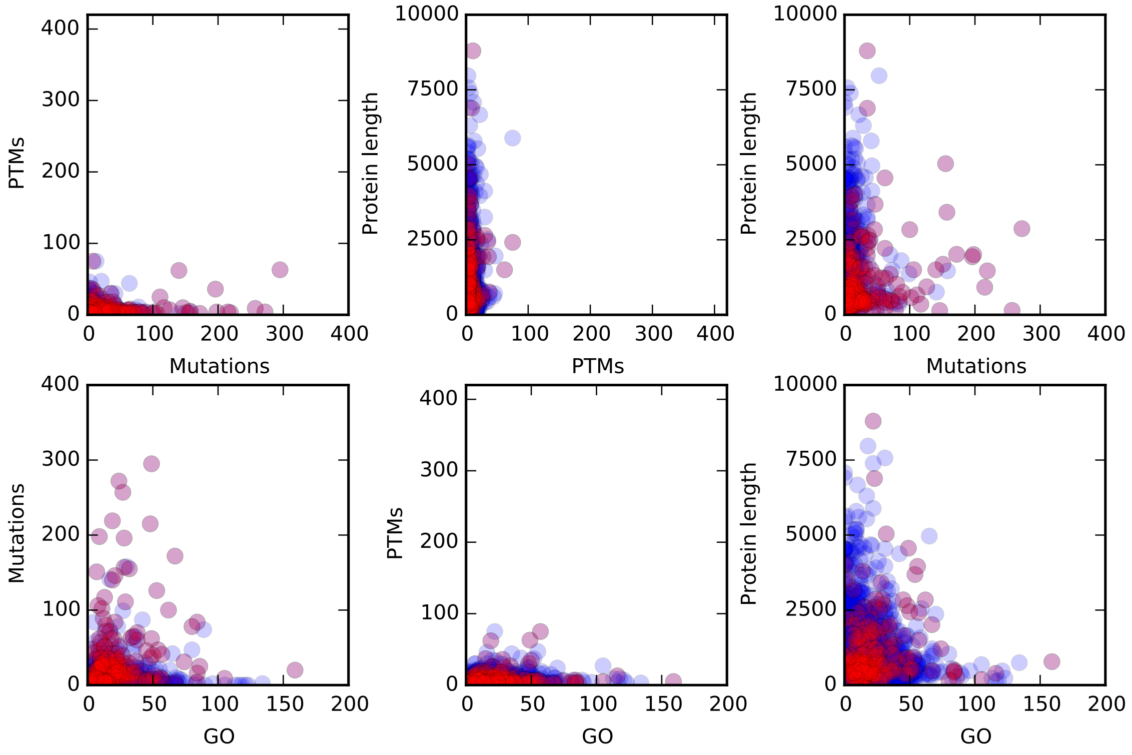

All

GO and Mutations have correlation 0.17 with pvalue of 8E-28
GO and N6AK have correlation 0.21 with pvalue of 3E-42
GO and Sequence have correlation -0.01 with pvalue of 4E-01
Mutations and N6AK have correlation 0.09 with pvalue of 9E-09
Mutations and Sequence have correlation 0.20 with pvalue of 8E-40
Sequence and N6AK have correlation 0.09 with pvalue of 3E-09

Pathogenic

GO and Mutations have correlation 0.15 with pvalue of 2E-02
GO and N6AK have correlation 0.06 with pvalue of 4E-01
GO and Sequence have correlation 0.09 with pvalue of 2E-01
Mutations and N6AK have correlation 0.05 with pvalue of 4E-01
Mutations and Sequence have correlation 0.20 with pvalue of 2E-03
Sequence and N6AK have correlation 0.14 with pvalue of 4E-02

UniProtKB: Ubiquitination

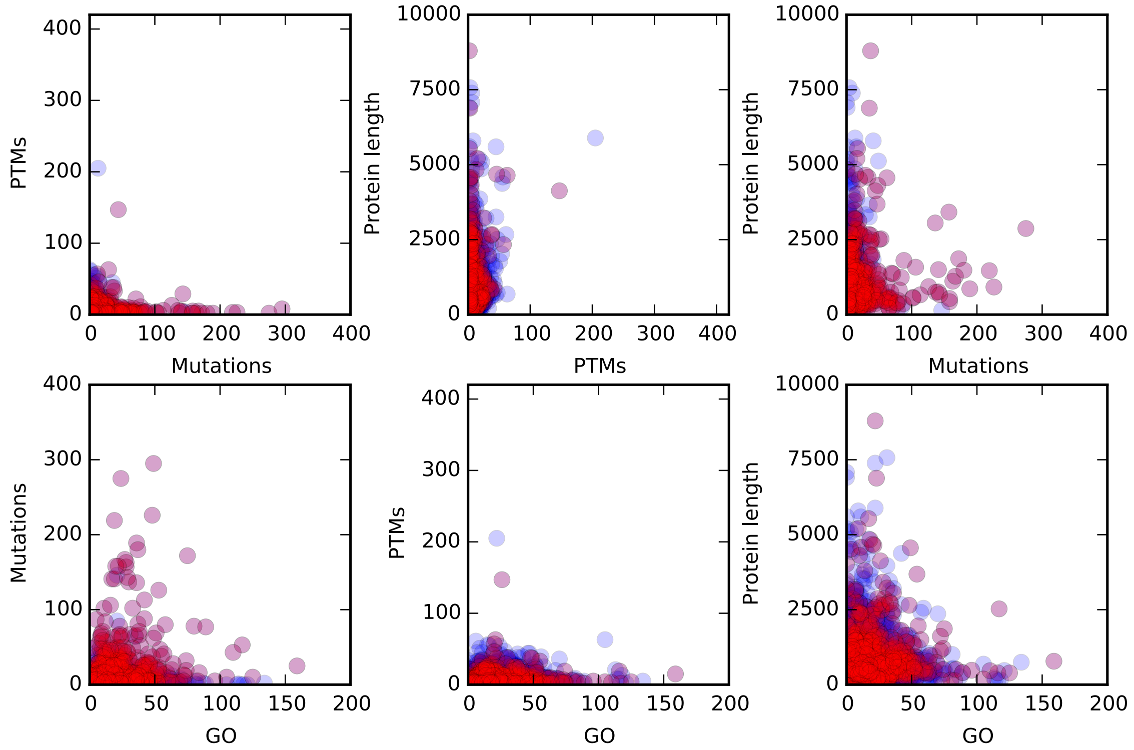

All

GO and Mutations have correlation 0.20 with pvalue of 1E-52
GO and Sequence have correlation 0.01 with pvalue of 6E-01
GO and Ubiq have correlation 0.23 with pvalue of 4E-67
Mutations and Ubiq have correlation 0.02 with pvalue of 8E-02
Mutations and Sequence have correlation 0.20 with pvalue of 5E-51
Sequence and Ubiq have correlation 0.17 with pvalue of 6E-40

Disease

GO and Mutations have correlation 0.22 with pvalue of 8E-11
GO and Sequence have correlation 0.02 with pvalue of 6E-01
GO and Ubiq have correlation 0.09 with pvalue of 8E-03
Mutations and Ubiq have correlation -0.02 with pvalue of 5E-01
Mutations and Sequence have correlation 0.17 with pvalue of 6E-07
Sequence and Ubiq have correlation 0.18 with pvalue of 3E-07

UniProtKB: Phosphotyrosine

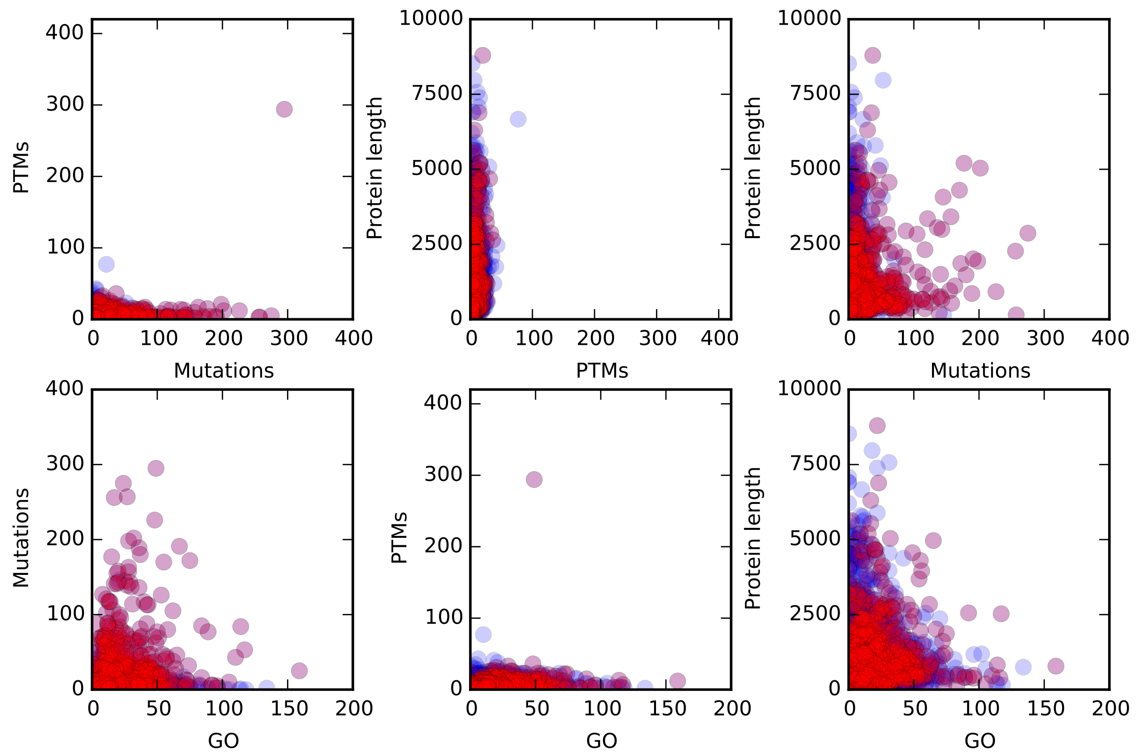

All

```
GO and Mutations have correlation   0.22 with pvalue of 2E-89
GO and Sequence have correlation    0.00 with pvalue of 7E-01
GO and pTyr have correlation  0.20 with pvalue of 3E-72
Mutations and pTyr have correlation       0.10 with pvalue of 4E-20
Mutations and Sequence have correlation   0.21 with pvalue of 2E-81
Sequence and pTyr have correlation  0.32 with pvalue of 9E-190
```

Disease

```
GO and Mutations have correlation   0.23 with pvalue of 1E-15
GO and Sequence have correlation    0.05 with pvalue of 1E-01
GO and pTyr have correlation  0.24 with pvalue of 2E-16
Mutations and pTyr have correlation       0.09 with pvalue of 1E-03
Mutations and Sequence have correlation   0.23 with pvalue of 3E-15
Sequence and pThr have correlation  0.54 with pvalue of 9E-89
```

UniProtKB:Phosphoserine

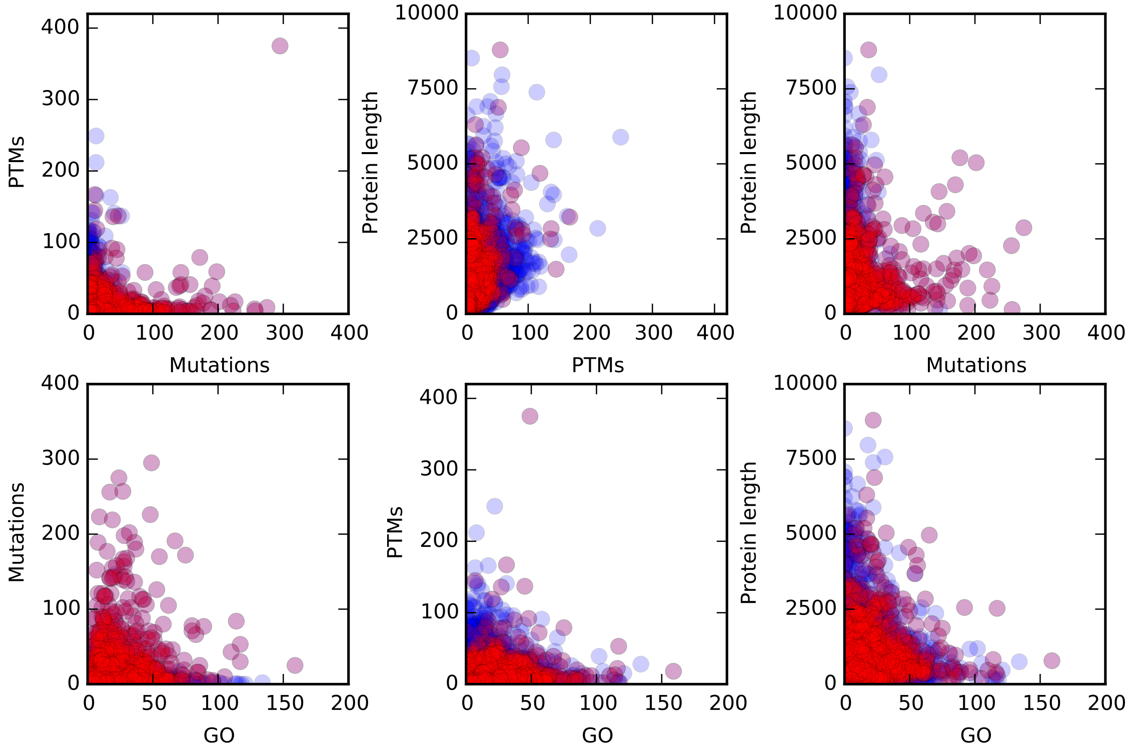

All
GO and Mutations have correlation 0.23 with pvalue of 6E-172
GO and Sequence have correlation 0.01 with pvalue of 2E-01
GO and pSer have correlation 0.12 with pvalue of 2E-48
Mutations and pSer have correlation 0.09 with pvalue of 2E-24
Mutations and Sequence have correlation 0.20 with pvalue of 8E-123
Sequence and pSer have correlation 0.46 with pvalue of 0E+00

Disease
GO and Mutations have correlation 0.22 with pvalue of 9E-20
GO and Sequence have correlation 0.04 with pvalue of 8E-02
GO and pSer have correlation 0.17 with pvalue of 2E-12
Mutations and pSer have correlation 0.10 with pvalue of 3E-05
Mutations and Sequence have correlation 0.22 with pvalue of 1E-19
Sequence and pSer have correlation 0.46 with pvalue of 1E-89

UniProtKB: Phosphothreonine

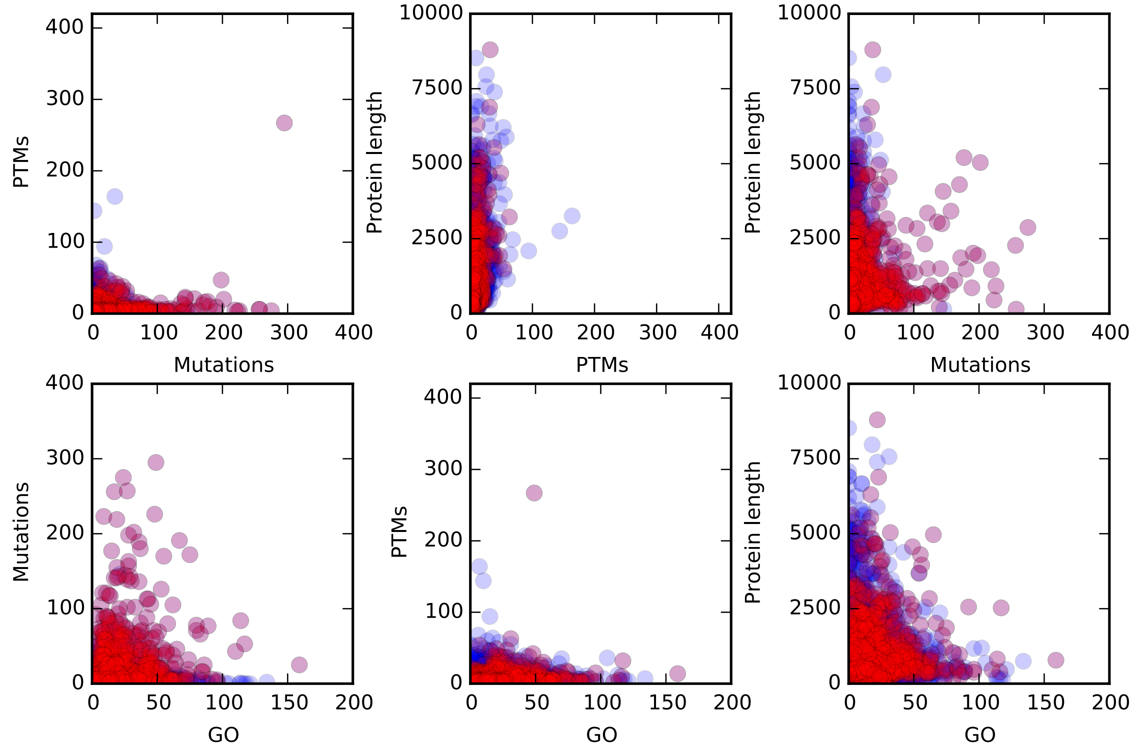

All
GO and Mutations have correlation 0.23 with pvalue of 2E-121
GO and Sequence have correlation -0.00 with pvalue of 8E-01
GO and pThr have correlation 0.14 with pvalue of 5E-43
Mutations and pThr have correlation 0.10 with pvalue of 1E-23
Mutations and Sequence have correlation 0.21 with pvalue of 4E-98
Sequence and pThr have correlation 0.44 with pvalue of 0E+00

Disease
GO and Mutations have correlation 0.24 with pvalue of 9E-18
GO and Sequence have correlation 0.02 with pvalue of 4E-01
GO and pThr have correlation 0.18 with pvalue of 1E-10
Mutations and pThr have correlation 0.09 with pvalue of 7E-04
Mutations and Sequence have correlation 0.23 with pvalue of 3E-17
Sequence and pThr have correlation 0.50 with pvalue of 5E-80

UniProtKB: N6-acetyllysine

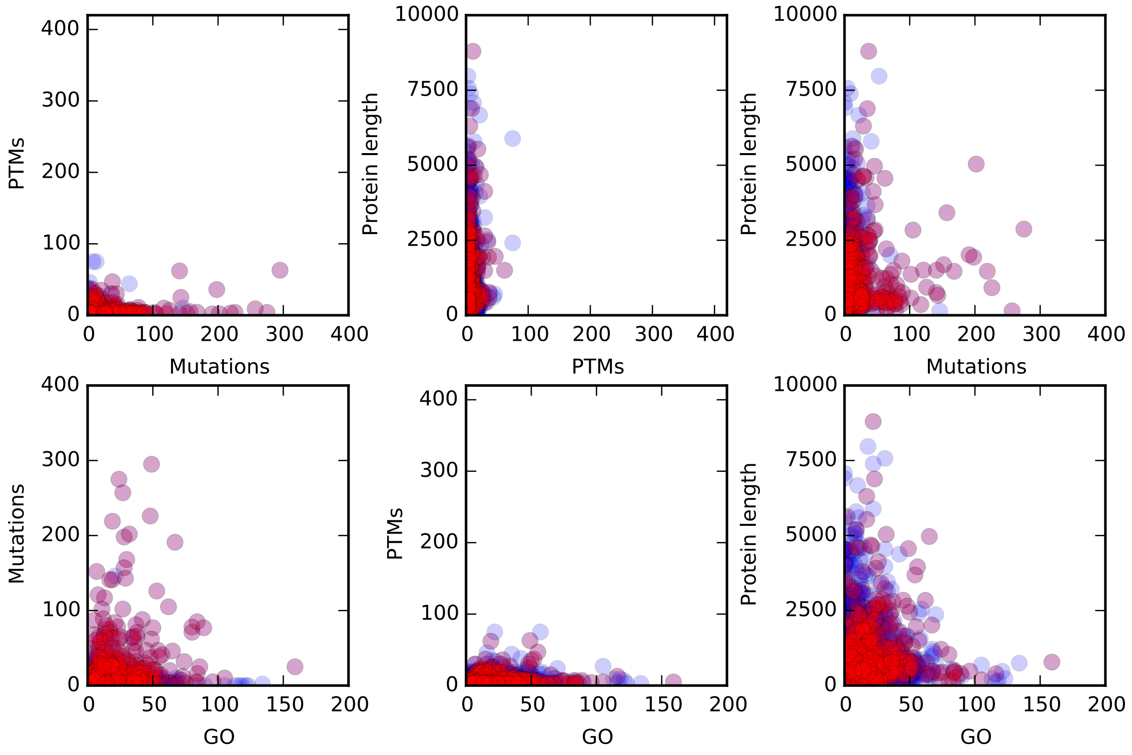

All
GO and Mutations have correlation 0.19 with pvalue of 5E-35
GO and Sequence have correlation -0.02 with pvalue of 2E-01
GO and N6AK have correlation 0.21 with pvalue of 2E-42
Mutations and N6AK have correlation 0.09 with pvalue of 3E-08
Mutations and Sequence have correlation 0.21 with pvalue of 2E-42
Sequence and N6AK have correlation 0.09 with pvalue of 5E-09

Disease
GO and Mutations have correlation 0.22 with pvalue of 2E-08
GO and Sequence have correlation 0.04 with pvalue of 3E-01
GO and N6AK have correlation 0.11 with pvalue of 4E-03
Mutations and N6AK have correlation 0.03 with pvalue of 5E-01
Mutations and Sequence have correlation 0.18 with pvalue of 3E-06
Sequence and N6AK have correlation 0.16 with pvalue of 6E-05
